# Overexpression of Wild-Type Human Alpha-Synuclein Causes Metabolism Abnormalities in Thy1-aSYN Transgenic Mice

**DOI:** 10.3389/fnmol.2018.00321

**Published:** 2018-10-02

**Authors:** Elodie Cuvelier, Mathieu Méquinion, Coline Leghay, William Sibran, Aliçia Stievenard, Alessia Sarchione, Marie-Amandine Bonte, Christel Vanbesien-Mailliot, Odile Viltart, Kevin Saitoski, Emilie Caron, Alexandra Labarthe, Thomas Comptdaer, Pierre Semaille, Hélène Carrié, Eugénie Mutez, Bernard Gressier, Alain Destée, Marie-Christine Chartier-Harlin, Karim Belarbi

**Affiliations:** ^1^UMR-S 1172, Centre de Recherche Jean-Pierre AUBERT Neurosciences et Cancer, Inserm, Centre Hospitalier Régional Universitaire de Lille, Université de Lille, Lille, France; ^2^UMR 894, Centre de Psychiatrie et Neurosciences, Inserm, Université Paris Descartes, Paris, France

**Keywords:** body weight, energy metabolism, insulin, leptin, mTOR, neurodegeneration, parkinsonism, transcription factor STAT3

## Abstract

Parkinson’s disease is a progressive neurodegenerative disorder characterized by loss of dopaminergic neurons, pathological accumulation of alpha-synuclein and motor symptoms, but also by non-motor symptoms. Metabolic abnormalities including body weight loss have been reported in patients and could precede by several years the emergence of classical motor manifestations. However, our understanding of the pathophysiological mechanisms underlying body weight loss in PD is limited. The present study investigated the links between alpha-synuclein accumulation and energy metabolism in transgenic mice overexpressing Human wild-type (WT) alpha-synuclein under the Thy1 promoter (Thy1-aSYN mice). Results showed that Thy1-aSYN mice gained less body weight throughout life than WT mice, with significant difference observed from 3 months of age. Body composition analysis of 6-month-old transgenic animals showed that body mass loss was due to lower adiposity. Thy1-aSYN mice displayed lower food consumption, increased spontaneous activity, as well as a reduced energy expenditure compared to control mice. While no significant change in glucose or insulin responses were observed, Thy1-aSYN mice had significantly lower plasmatic levels of insulin and leptin than control animals. Moreover, the pathological accumulation of alpha-synuclein in the hypothalamus of 6-month-old Thy1-aSYN mice was associated with a down-regulation of the phosphorylated active form of the signal transducer and activator of transcription 3 (STAT3) and of Rictor (the mTORC2 signaling pathway), known to couple hormonal signals with the maintenance of metabolic and energy homeostasis. Collectively, our results suggest that (i) metabolic alterations are an important phenotype of alpha-synuclein overexpression in mice and that (ii) impaired STAT3 activation and mTORC2 levels in the hypothalamus may underlie the disruption of feeding regulation and energy metabolism in Thy1-aSYN mice.

## Introduction

Parkinson’s disease (PD) is the most common movement neurodegenerative disorder in elderly adults. It is characterized by a progressive degeneration of dopaminergic neurons in the *substantia nigra* and by the pathological accumulation of intraneuronal aggregated and hyperphosphorylated alpha-synuclein in Lewy bodies (Bridi and Hirth, 2018). Missense mutations and multiplication of the gene encoding alpha-synuclein *SNCA* (synuclein, alpha [non-A4 component of amyloid precursor]) were identified as genetic abnormalities associated with rare familial forms of PD (Polymeropoulos et al., 1997; Singleton et al., 2003; Chartier-Harlin et al., 2004; Ibanez et al., 2004). Polymorphisms regulating *SNCA* levels were subsequently associated with sporadic PD (Maraganore et al., 2006; Simon-Sanchez et al., 2009), supporting that alpha-synuclein level is instrumental in most forms of the disease. Dopamine deficit at the striatum -e.g., the striatal area innervated by the *substantia nigra*- is the main factor leading to bradykinesia, resting tremor, rigidity and postural instability. It is generally accepted that these motor symptoms appear only after a substantial proportion of dopaminergic neurons are lost (Bezard et al., 2001).

The motor features of PD can be preceded, sometimes for several years, by non-motor symptoms such as olfactory deficits, sleep disorders, depression and autonomic dysfunction (Schapira et al., 2017). Increasing evidence suggests that unintended body weight change is also a significant feature of PD symptomatology. Weight loss, primarily due to fat rather than muscle loss (Markus et al., 1993), has been frequently documented in PD patients (Chen et al., 2003; Cheshire and Wszolek, 2005; Uc et al., 2006; van der Marck et al., 2012). The majority of patients have lower body mass than control individuals at diagnosis (Sharma and Lewis, 2017). PD patients are moreover four times more likely to lose body mass than healthy elderly individuals (Chen et al., 2003; Cheshire and Wszolek, 2005; Uc et al., 2006; van der Marck et al., 2012; Cumming et al., 2017). Body mass loss during the course of the disease has been associated with poorer clinical outcomes and rapid disease progression (Lorefalt et al., 2004; van der Marck et al., 2012; Sharma and Vassallo, 2014; Pak et al., 2018), suggesting that it could be of prognostic significance for PD severity (Cumming et al., 2017; Sharma and Lewis, 2017; Pak et al., 2018). Thus, to address the links between weight loss and PD is quite necessary (Ma et al., 2018).

Body weight is governed by energy intake and energy expenditure (EE), which are tightly controlled as peripheral hormonal signals integrate in the hypothalamus to regulate food intake and energy outgo (Schwartz et al., 2000). Reduced energy intake, secondary to motor or non-motor symptoms (i.e., hyposmia, gastrointestinal disturbance, depression) has been proposed as a factor contributing to weight loss in PD (Ma et al., 2018). However, other studies showed that mild to moderate PD patients have the same nutritional status compared to controls (Fereshtehnejad et al., 2014) or that weight loss can occur in PD despite an increased energy intake (Chen et al., 2003; Lorefalt et al., 2004). Conflicting results also exist regarding EE, with a study suggesting that increased EE could contribute to weight loss in PD (Markus et al., 1992) while others showed that the total daily EE was not higher in patients with weight loss compared with patients without weight loss (Delikanaki-Skaribas et al., 2009) and healthy controls (Jorgensen et al., 2012). Central regulatory hypothalamic mechanisms in weight disturbance in PD has recently attracted much attention in part due reports of body weight gain after deep brain stimulation of subthalamic nuclei (Bannier et al., 2009; Aiello et al., 2017). Moreover, changes in plasmatic concentrations of hormones regulating energy balance such as leptin occur in PD patients with weight loss (Fiszer et al., 2010). However, our basic understanding of the pathophysiological mechanisms underlying body weight change in PD remains limited. In particular it is not known whether alpha-synuclein accumulation that is central in PD impacts on energy intake/expenditure, levels of peripheral hormones or changes in global function of the hypothalamus.

In the present study, we determined the metabolic phenotype of the well-established Thy1-aSYN mouse model of PD. Thy1-aSYN transgenic mice overexpress full-length Human wild-type (WT) alpha-synuclein under the murine Thy-1 (thymus cell antigen 1, theta) promoter (Thy1-aSYN mice) (Chesselet et al., 2012). We measured the evolution of body mass from weaning to 12 months of age and assessed in 6-month-old (an age preceding any severe motor deficits) animals food intake, spontaneous activity, EE, glucose, and insulin tolerance, as well as alpha-synuclein, insulin, and leptin in the plasma and related signaling pathways in the hypothalamus.

## Materials and Methods

### Animal Procedures

This research was conducted in accordance with the European Union standards for the care and use of laboratory animals and approved by the Nord/Pas-de-Calais Ethical Committee CEEA N°75 (authorization n° O535.02). Thy1-aSYN mice (on a C57Bl6/DBA2 background) were provided by Pr. Marie-Françoise Chesselet (University of California Los Angeles, CA, United states) with the agreement of Prof. Eliezer Masliah (University of California San Diego, CA, United States) and a colony was established in Lille animal facility by breeding transgenic females with WT males (Charles Rivers). Animals were genotyped by PCR from tail DNA samples. Thy1-aSYN and WT littermates male mice were used in the study. Animals were maintained in standard animal cages under specific pathogen-free conditions (12/12 h light/dark cycle; 22°C; grouped housed), with *ad libitum* access to water and standard laboratory chow (RM1A; 14.8 MJ/kg; Special Diets Services). Body weights were measured for different animals housed in our colony, including but not limited to those euthanized for further analyses, at weaning (WT: *n* = 17, Thy1-aSYN: *n* = 17), 2 months (WT: *n* = 17, Thy1-aSYN: *n* = 17), 3 months (WT: *n* = 27, Thy1-aSYN: *n* = 25), 6 months (WT: *n* = 33, Thy1-aSYN: *n* = 29), 9 months (WT: *n* = 10, Thy1-aSYN: *n* = 9) and 12 months (WT: *n* = 10, Thy1-aSYN: *n* = 7) of age. Further behavioral and metabolic characterizations were performed on 6-month-old animals (WT: *n* = 22, Thy1-aSYN: *n* = 19) or 3-month-old animals (WT: *n* = 16, Thy1-aSYN: *n* = 13). Beam test was performed on 6-month-old animals and lasted 3 days, as described below. The following week, metabolic analyses were carried out using metabolic cages (see below) and glucose tolerance and insulin sensitivity tests were performed (see below). The body compositions were assessed on 12 WT and 10 Thy1-aSYN 6-month-old mice. For blood and plasma analyses, mice were fasted during 6 h before being euthanized. Trunk blood samples were gathered in heparinized tubes and centrifuged at 425 g for 10 min at 4°C. Plasma supernatant was aliquoted and stored at -70°C until assayed. Brains were rapidly removed, hypothalamus dissected out at 4°C and stored at -70°C until use. Inguinal and gluteal adipose tissue were weighted as subcutaneous adipose tissue. The same experimenter dissected all samples for consistency.

### Challenging Beam Traversal Test

Motor performance and coordination were measured using a challenging beam traversal procedure (Fleming et al., 2013). Briefly, animals were trained for 2 days in the afternoon to traverse the length of a beam toward their home cage. On the third day, a mesh grid (1 cm squares) was positioned 1 cm above the beam. Mice were then videotaped for a total of five trials. An experimenter blind to genotype watched and rated the videotapes for errors, number of steps made by each animal, and time to traverse for all five trials. An error was counted when, during a forward movement, a limb slipped through the grid and was visible between the grid and the beam surface (Fleming et al., 2013).

### Body Composition

Body composition was determined using an “*in vivo* Micro-CT Scanner for Small Lab Animals” (LaTheta LCT-100, Hitachi Aloka Medical Ltd.). Mice were anesthetized by intraperitoneal injection of ketamine (100 mg/kg) and xylazine (20 mg/kg) mix and about 60 CT slices per mouse were made at 500 μm intervals between shoulders and posterior legs. Slices were analyzed by Aloka software for fat (visceral and subcutaneous) mass, lean mass, and for bone mineral density in the spinal cord. The fat ratio was calculated by the following formula: total fat mass/(total fat mass + lean mass) × 100.

### Metabolism, Ambulatory Activity and Energy Expenditure Monitoring

Mice were individually housed in a LabMaster-CaloSys-Calorimetry System (TSE Systems). Animals were placed 3 days in chambers and only the last 24 h were monitored. The locomotor activity was derived from the number of beam breaks (infrared light-beam frame ActiMot2; x- and z- axes sensors). The system measured the volume of O_2_ consumed and the volume of CO_2_ produced, over a 15 min period, 4 times per hour. These values were averaged to determine the rate of O_2_ consumed (VO_2_) and CO_2_ produced (VCO_2_). The EE, the respiratory exchange ratio (RER), and the fatty acid oxidation (FA) were calculated using the following equations RER = VCO_2_ / VO_2_; EE (kcal/h) = (3.815 + 1.232 × RER) × VO_2_) × 1000, FA oxidation (kcal/h) = EE × ((1-RER)/0.3) (Mequinion et al., 2015). Values for RER range between 1.0 to 0.7, with pure carbohydrate oxidation having a value of 1.0 and pure fat oxidation having a value of 0.7.

### Food Intake Monitoring/Meal Pattern Analysis

Metabolic cages are equipped with food and drink high precision sensors of 0.01 g and 0.01 ml resolution respectively (TSE Systems, GmbH). Mice had free access to food and water during the whole recording period. Here a meal was defined as a consumption of at least 0.03 g of food separated from the next feeding episode by at least 10 min, as previously described (Hassouna et al., 2013). For each mouse, inter-meal interval, meal duration, meal size and meal rate in dark and light phases were assessed.

### Intraperitoneal Glucose Tolerance and Insulin Sensitivity

Intraperitoneal glucose tolerance and insulin sensitivity tests were performed at 2 p.m., after 6 h of fasting. D (+) glucose (1 g/kg in saline; Sigma-Aldrich) or insulin (0.75 units/kg in saline; NovoRapid FlexPen^®^) were injected intraperitoneally. Blood glucose concentration was measured using a OneTouch Vita^®^ meter (Lifescan) 0, 15, 30, 60, 90 and 120 min following injection.

### Blood Glucose and Plasmatic Alpha-Synuclein, Insulin and Leptin Levels

Blood glucose levels were quantified after 6 h of fasting using a OneTouch Vita^®^ meter (Lifescan). Plasma Human alpha-synuclein levels were measured using the Human alpha-Synuclein Kit (Meso Scale Discovery) according to the manufacturer’s instructions. Intraassay coefficient of variation was < 14%. Plasma insulin was assayed using the Mercodia Ultrasensitive Mouse Insulin ELISA (Mercodia). Intra- and interassay coefficients of variation were <3.4 and <3.0%, respectively. Plasma leptin was measured using the Quantikine^®^ ELISA kit (R&D Systems). Intra- and interassay coefficients of variation were <4.5 and <4.7%, respectively. All the samples were analyzed in duplicate.

### Western-Blot Analyses

Tissue was homogenized in 300 μl RIPA buffer (Thermo Scientific) containing 0.5% (w/v) CHAPS (Sigma-Aldrich), protease and phosphatase inhibitors (Complete and PhosSTOP, Roche), using a glass/teflon potter homogenizer (30 strokes), sonicated and let under agitation for 1 h at 4°C. Lysates were centrifuged at 12000 × *g* for 20 min at 4°C. The supernatant was removed and stored at -20°C. Proteins were quantified using the BCA system (Pierce). Protein samples were prepared in reducing conditions (NuPage sample buffer with sample reducing agent, Invitrogen) and heated at 95°C for 10 min. Then, 10 μg of proteins were separated on a NuPAGE Novex gel (Invitrogen) and transferred to a nitrocellulose or PVDF membrane, for chemiluminescent or fluorescent western-blotting respectively. Membranes were saturated in 5% non-fat dry milk in TNT or 5% Bovine Serum Albumine in Tris–NaCl-Tween buffer and incubated with appropriate primary antibodies allowing detection of actin (Sigma-Aldrich A5441, 1/50000), Akt (Cell Signaling 9272, 1/5000), phospho-Akt (Ser473) (Cell Signaling 12694, 1/2500), Human and murine alpha-synuclein (BD Bioscience 610787; immunogen: Rat synuclein-1 aa. 15-123; 1/10000), phospho-alpha-Synuclein (Ser129) (Cell Signaling 23706; immunogen: synthetic phosphopeptide corresponding to residues surrounding Ser129 of Human alpha-synuclein protein; 1/1000), aggregated alpha-synuclein (Merck MABN389; immunogen: Keyhole limpet hemocyanin-conjugated linear peptide corresponding to Human aggregated alpha-synuclein; 1/2000), IRS-1 (Cell Signaling 2382, 1/1000), phospho-IRS1 (Tyr608) (Merck 09-432, 1/5000), LC3B (Abcam ab48394, 1/2500), mTOR (Cell Signaling 2972, 1/1000), phospho-mTOR (Ser2448) (Cell Signaling 2971, 1/2500), p62 (BD Bioscience 610498, 1/5000), Stat3 (Cell Signaling 9139, 1/2500), phospho-Stat3 (Tyr705) (Cell Signaling 9131, 1/1000), Raptor (Cell Signaling 2280, 1/5000) and Rictor (Cell Signaling 2114, 1/5000). Signals were revealed with horseradish peroxidase conjugated secondary antibodies (Life technologies) and chemiluminescence (ECL Prime Western Blotting Detection Reagent, Amersham Biosciences) using Amersham Imager 600 (GE Healthcare), or with fluorescent secondary antibodies (Life technologies) using Typhoon FLA 9500 GoldSeal (GE Healthcare). Two to eight replicates of each western-blot were performed. Quantification of protein bands densitometry was carried out using ImageJ software version 1.51 w (NIH). Results are normalized to actin levels.

### Statistics

The difference in body mass between genotypes was compared across time using a Kruskal–Wallis test followed by Mann–Whitney *U*-test on individual time period. A Mann–Whitney *U*-test was used to compare WT and Thy1-aSYN mice for a given phase or light and dark phases for a given phenotype. Unless otherwise noted, values in the figures and text are mean ± standard error of the mean (SEM). Values of *p* < 0.05 are considered to be statistically significant. Data were analyzed and graphs were plotted by GraphPad Prism^®^ software version 6.05.

## Results

### Thy1-aSYN Mice Show Impaired Motor Performance and Coordination in Challenging Beam Traversal

**FIGURE 1:**
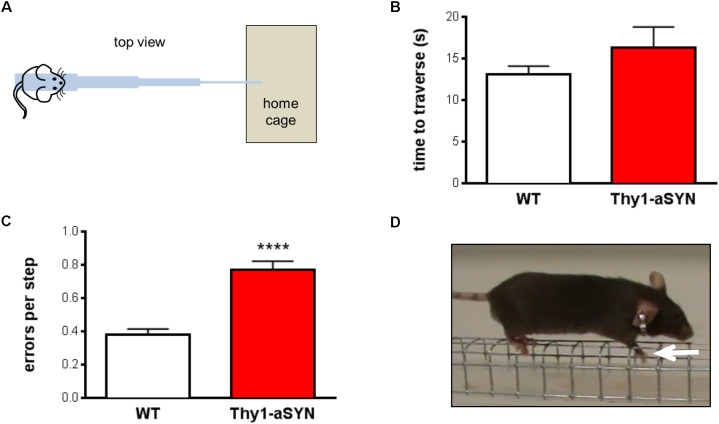
Motor performance and coordination in 6-month-old mice. **(A)** Schematic representation of the challenging beam traversal procedure used to assess motor performance. **(B)** Time to traverse did not differ between experimental groups. **(C)** Thy1-aSYN mice made more errors per step compared to WT mice. **(D)** Photograph of a mouse making an error on the grid while traversing the beam (white arrow: forelimb slips through the grid). Mean of five trials. Mann–Whitney *U*-test. ^∗∗∗∗^*p* < 0.0001 vs. WT mice.

Six-month-old WT and Thy1-aSYN mice were tested in the challenging beam traversal test (**Figure 1A**). Time to traverse, number of steps and error per steps were analyzed. Regarding time to traverse, Mann–Whitney *U*-test showed no significant difference between WT and transgenic mice (*p* = 0.67) (**Figure 1B**). Similarly, the number of steps did not differ between WT and transgenic mice (*p* = 0.28; data not shown). Analysis of the number of errors per step indicated that transgenic animals made significantly more errors compared to WT controls (0.77 ± 0.05 vs. 0.38 ± 0.03; *p* < 0.0001) (**Figures 1C,D**). Therefore, our results show that 6-month-old Thy1-aSYN display impairments in the challenging beam traversal test, confirming the deficits of this model in challenging motor tests (Fleming et al., 2004; Chesselet et al., 2012).

### Thy1-aSYN Mice Show Reduced Body Weight Gain Over Time

**FIGURE 2:**
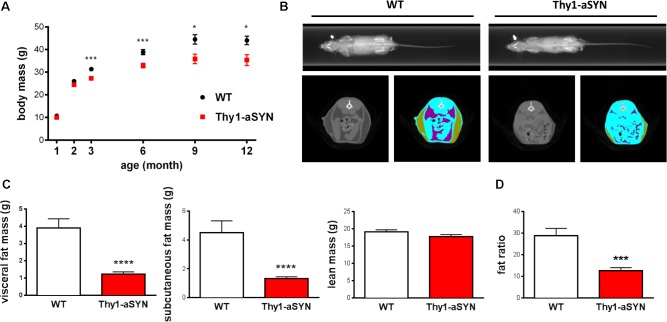
Body weight gain and body composition analysis. **(A)** Thy1-aSYN mice show reduced weight-gain compared to WT littermates from 3 months of age. **(B)** Representative Micro-CT Scans obtained from 6-month-old WT and Thy1-aSYN mice showing selected area of lean (blue), subcutaneous fat (green), visceral fat (purple) tissues. **(C)** Both visceral and subcutaneous fat masses were decreased in 6-month-old Thy1-aSYN mice compared to WT mice, with no change in lean mass. **(D)** Mass of subcutaneous adipose tissue collected at euthanasia was decreased in 6-month-old Thy1-aSYN mice compared to WT mice. Mann–Whitney *U*-test on individual time epochs and for body composition analyses; ^∗^*p* < 0.05, ^∗∗∗^*p* <0.01, and ^∗∗∗∗^*p* < 0.0001 vs. WT mice.

Wild-type and transgenic mice were weighed at 21 days (weaning), 2, 3, 6, 9, and 12 months of age. Kruskal–Wallis analysis showed that both WT and transgenic mice gained weight as they aged (*p* < 0.0001 for both genotype). Mann–Whitney *U*-test on individual time periods revealed that Thy1-aSYN mice weighed significantly less than WT mice from 3 months of age (27.25 ± 0.92 vs. 31.31 ± 0.94 g; *p* < 0.001). Difference increased over time, so that at 12 months of age WT mice reached 42.72 ± 2.13 g, while Thy1-aSYN mice weighted 35.30 ± 2.44 g (*p* < 0.05) (**Figure 2A**). Therefore, our results suggest a slower body weight gain of Thy1-aSYN transgenic mice compared to WT littermates. Next characterizations were conducted on 6-month-old transgenic Thy1-aSYN and WT mice (body mass: Thy1-aSYN: 32.84 ± 1.03 vs. WT: 38.82 ± 1.13 g; *p* < 0.001).

### Thy1-aSYN Mice Have Reduced Body Fat Compared to WT Mice

To explore whether lower body mass of Thy1-aSYN compared to WT mice was attributable to change in body composition, X-ray CT Scan imaging was used to evaluate lean and fat masses in anesthetized 6-month-old mice (**Figure 2B**). Data analyses revealed no significant difference in lean mass between transgenic and WT mice (Thy1-aSYN: 17.69 ± 0.63 vs. WT: 19.15 ± 0.54; *p* = 0.11). In contrast, transgenic mice showed a decrease in the total fat mass (2.54 ± 0.26 vs. 8.39 ± 1.35 g; *p* < 0.0001), visceral fat mass (1.22 ± 0.14 vs. 3.90 ± 0.53 g; *p* < 0.0001), and subcutaneous fat mass (1.32 ± 0.125 vs. 4.49 ± 0.82 g; *p* < 0.0001) compared to WT littermates (**Figure 2C**). This was accompanied by a decreased fat ratio (Thy1-aSYN: 12.67 ± 1.36 vs. WT: 28.88 ± 3.29%; *p* = 0.0001). Bone mineral density was also calculated and showed no difference between transgenic and WT animals (data not shown). At euthanasia, the weight of subcutaneous adipose tissue (consisting of inguinal and gluteal adipose tissues) was lower in Thy1-aSYN mice compared to that of WT mice (121.1 ± 10.58 vs. 447.4 ± 77.32 mg; *p* < 0.001) (**Figure 2D**). Therefore, both CT Scan imaging and subcutaneous fat weighting show that Thy1-aSYN mice have a reduced fat accumulation.

### Thy1-aSYN Mice Display Reduced Food Intake and Altered Meal Pattern

**FIGURE 3:**
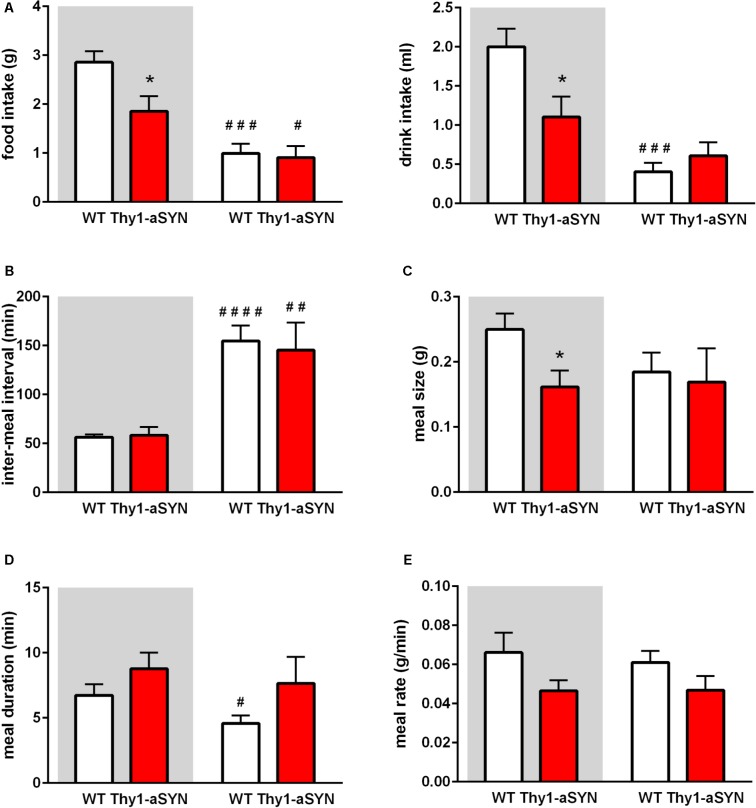
Food intake and meal pattern of 6-month-old mice. **(A)** Thy1-aSYN mice consumed less food and water compared to WT mice during the dark phase. **(B)** Inter-meal interval were more elevated during the light phase than during the dark phase, regardless of the genotype. **(C)** Meal size was decreased in Thy1-aSYN mice compared to WT mice during the dark phase. **(D)** Meal duration and **(E)** Meal rate analyses show no statistically significant difference between genotypes, although Thy1-aSYN mice overall tended to show longer meal duration and decreased meal rate compared to WT mice during the dark phase. Mann–Whitney *U*-test. ^#^*p* < 0.05, ^##^*p* < 0.01, ^###^*p* < 0.001, and ^####^*p* < 0.0001 vs. dark cycle; ^∗^*p* < 0.05 vs. WT mice. White and gray backgrounds represent light and dark phases, respectively.

To better understand the cause of the lower body weight and body fat in transgenic mice, we examined their food intake, ambulatory activity and EE using the LabMaster-CaloSys-Calorimetry system. Transgenic mice consumed 35% less food than their control littermates during the dark cycle (1.85 ± 0.31 vs. 2.86 ± 0.225 g; *p* < 0.05). This was accompanied by a lower consumption of water (1.11 ± 0.26 vs. 2.00 ± 0.23 ml; *p* < 0.05) (**Figure 3A**). Further analyses were performed to compare the inter-meal interval, meal size, meal duration and meal rate. Inter-meal interval analysis revealed no difference between transgenic and WT mice and both genotypes showed a more elevated inter-meal interval during the light phase than during the dark phase (**Figure 3B**). Measurement of the meal size showed that it was decreased in Thy1-aSYN mice compared to WT mice during the dark phase (0.162 ± 0.025 vs. 0.250 ± 0.025 g; *p* < 0.05) (**Figure 3C**). Thy1-aSYN mice also tended to show a longer meal duration and a decreased meal rate compared to WT mice, although these differences did not reach statistical significance (**Figures 3D,E**). It however has to be noted that the meal duration was statistically shorter during the light cycle compared to the dark cycle for WT animals (4.58 ± 0.59 vs. 6.73 ± 0.85 min; *p* < 0.05), but this was not the case for transgenic animals (**Figure 3D**).

### Thy1-aSYN Mice Show Increased Activity and Altered Energy Metabolism

**FIGURE 4:**
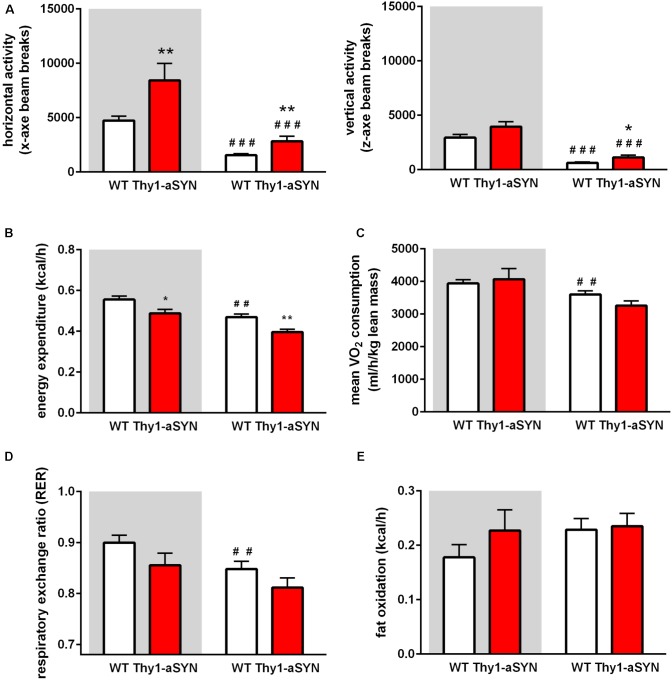
Spontaneous activity and energy metabolism in 6-month-old mice. **(A)** Horizontal (x-axe) and vertical activity (z-axe) of Thy1-aSYN mice and WT mice. **(B)** Energy expenditure was decreased in Thy1-aSYN mice compared to WT mice, both during the dark cycle and light phases. **(C)** Oxygen consumption, **(D)** RER and **(E)** Fat oxidation of Thy1-aSYN and WT mice. Mann–Whitney *U*-test. ^##^*p* < 0.01, ^###^*p* < 0.001 vs. dark cycle; ^∗^*p* < 0.05 and ^∗∗^*p* < 0.01 vs. WT mice. White and gray backgrounds represent light and dark phases, respectively.

The monitoring of locomotor activity showed that the horizontal ambulatory activity (x-axis beam breaks counts; e.g., locomotion) of Thy1-aSYN mice was significantly higher than that of WT mice both during the dark cycle (8425 ± 1552 vs. 4731 ± 401 breaks; *p* < 0.05) and during the light cycle (2828 ± 446 vs. 1558 ± 132 breaks; *p* = *p* < 0.01). Likewise, the vertical activity (z-axis beam breaks counts; e.g., rearing or jumping) was significantly increased during the light cycle (1128 ± 218 vs. 620 ± 101 breaks; *p* < 0.05) (**Figure 4A**). The measurement of the EE using the LabMaster-CaloSys-Calorimetry system showed that EE was decreased in Thy1-aSYN mice compared to WT mice, both during the dark cycle (0.49 ± 0.02 vs. 0.56 ± 0.02 kcal/h; *p* < 0.05) and during the light cycle (0.40 ± 0.01 vs. 0.47 ± 0.02 kcal/h; *p* < 0.01) (**Figure 4B**). Body composition influences energy metabolism, and there is no consensus on the best normalization tool to use when expressing VO_2_ or VCO_2_ (Butler and Kozak, 2010; Kaiyala et al., 2010). In our study, we observed no statistically significant change in oxygen consumption or carbon dioxide production between transgenic and WT mice, when normalized to either lean mass or total body weight (**Figure 4C** and data not shown). However, our results show that oxygen consumption was decreased during the light cycle compared to the dark cycle for WT animals (3604 ± 110 vs. 3940 ± 115 ml/h/kg lean mass; *p* < 0.01), while this was not the case for the transgenic animals (**Figure 4C**). Similarly, the RER was significantly decreased during the light cycle compared to the dark cycle in WT animals (0.848 ± 0.015 vs 0.900 ± 0.014; *p* < 0.01) but no such difference was observed in Thy1-aSYN mice (**Figure 4D**). Altogether these data suggest that temporal rhythms in energy metabolism may be deregulated in Thy1-aSYN mice. Finally, as the Thy1-aSYN transgenic mice have lower body fat mass than WT mice, we calculated the fat oxidation to better evaluate their use of fat as intrinsic energy source. Our results did not reveal significant difference for this parameter between genotypes (dark cycle: Thy1-aSYN: 0.23 ± 0.04 vs. WT: 0.18 ± 0.02 kcal/h; *p* = 0.485; light cycle: Thy1-aSYN: 0.235 ± 0.02 vs. WT: 0.23 ± 0.02; *p* = 0.74) (**Figure 4E**).

### Thy1-aSYN Mice Have Decreased Insulin and Leptin Plasma Levels

**FIGURE 5:**
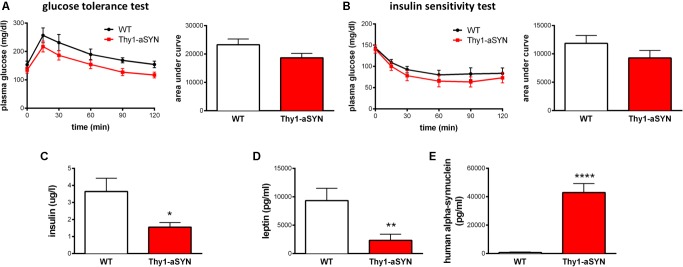
Glucose homeostasis and plasma levels of alpha-synuclein, insulin and leptin in 6-month-old animals. Thy1-aSYN and WT mice showed no significant difference in glycemic response patterns following intraperitoneal injection of **(A)** glucose or **(B)** insulin. Thy1-aSYN mice displayed **(C)** elevated alpha-synuclein levels, **(D)** low insulin and **(E)** low leptin plasma levels compared to WT mice. Mann–Whitney *U*-test; ^∗^*p* < 0.05, ^∗∗^*p* < 0.01, and ^∗∗∗∗^*p* < 0.0001 vs. WT mice.

Given the change in body weight, fat mass, food consumption, spontaneous activity and EE, we first examined blood glucose levels after 6 h of fasting and observed no significant difference between Thy1-aSYN and WT mice (TR: 160.0 ± 9.75 vs. WT: 172.5 ± 8.32 mg/dl; *p* = 0.4732). To assess whole-body glucose homeostasis, we next performed intraperitoneal glucose tolerance test and insulin sensitivity test on these mice. We detected no differences in the response patterns, with comparable area under the curve for Thy1-aSYN and WT mice in both tests (**Figures 5A,B**). We also measured the plasma levels of insulin and leptin and showed decreased levels of these two hormones in Thy1-aSYN mice compared to WT mice (insulin: 1.56 ± 0.27 vs. 3.64 ± 0.78 μg/l; *p* < 0.05; leptin: 2331 ± 1103 vs. 9348 ± 2163 pg/ml; *p* < 0.01) (**Figures 5C,D**). Finally, the presence of Human alpha-synuclein was detected in the plasma of transgenic mice but not in the plasma of WT mice (42960 ± 6305 pg/ml; *p* < 0.0001 vs. WT mice) (**Figure 5E**).

### Thy1-aSYN Mice Display Decreased STAT3 Phosphorylation and Rictor Level in the Hypothalamus

**FIGURE 6:**
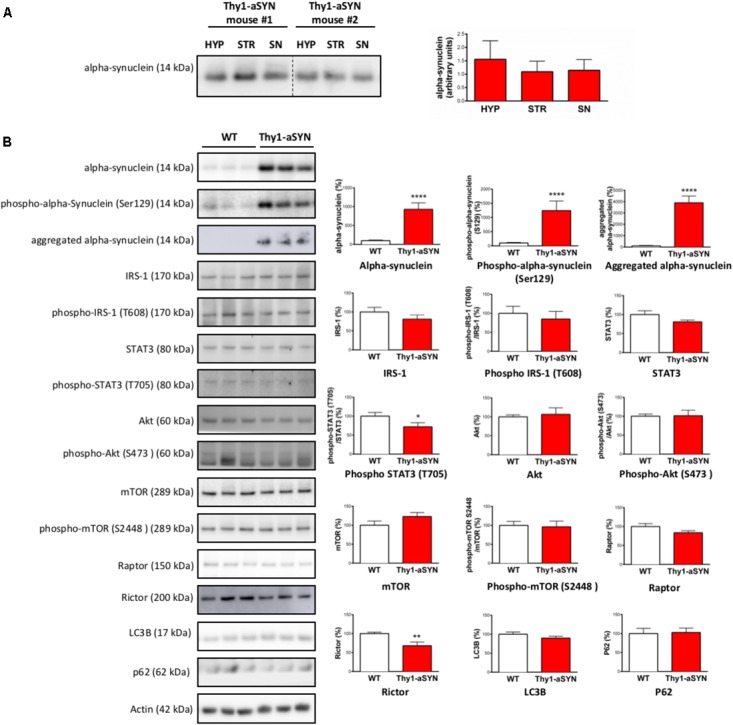
Western blot analysis of hypothalamus protein extracts from 6-month-old Thy1-aSYN and WT mice. **(A)** Alpha-synuclein was expressed at a comparable level in the hypothalamus, the *substantia nigra* and the striatum of 6-month-old Thy1-aSYN mice (Thy1-aSYN: *n* = 4). **(B)** The left images show representative bands for each target protein, and the results are quantified in the right graphs (WT: *n* = 12, Thy1-aSYN: *n* = 10). Thy1-aSYN mice displayed increased levels of total alpha-synuclein, alpha-synuclein phosphorylation at serine 129 and aggregation of alpha-synuclein, as well as decreased phosphorylation at tyrosine 705 of the transcription factor STAT3 and decreased levels of Rictor. Mann–Whitney *U*-test; ^∗^*p* < 0.05, ^∗∗^*p* < 0.01, and ^∗∗∗∗^*p* < 0.0001 vs. WT mice.

The control of feeding and EE is centrally regulated by the hypothalamus using the information originating from peripheral organs. To determine whether alterations in this brain region could contribute to the metabolic abnormalities in Thy1-aSYN mice, we first evaluated the accumulation of alpha-synuclein in the hypothalamus in our model. We found that alpha-synuclein, detected with an antibody that recognizes both mouse and Human proteins, was expressed in the hypothalamus at a level comparable to that observed in the substantia nigra and the striatum in 6-month-old Thy1-aSYN mice (**Figure 6A**). Further analyses showed that levels of total alpha-synuclein, alpha-synuclein phosphorylation at serine 129 and aggregated alpha-synuclein were higher in the hypothalamus of Thy1-aSYN mice compared to WT mice (928.5 ± 169.7 vs. 100 ± 17.38%; *p* < 0.0001; 1243.1 ± 335.8 vs. 100 ± 21.36%; *p* < 0.0001 and 4133.72 ± 638.6 vs. 100 ± 38.56%; *p* < 0.0001, respectively) (**Figure 6B**). We next compared the expression and activation of receptors and protein complexes known to be critically implicated in the regulation of energy balance and metabolism by the hypothalamus. Western blot of total and active phosphorylated forms of IRS-1 and of STAT3 showed that phosphorylation of STAT3 at tyrosine 705 was decreased in the hypothalamus of 6-month-old Thy1-aSYN mice compared to WT mice (71.84 ± 10.51 vs. 100 ± 9.70%; *p* < 0.05) (**Figure 6B**). We also compared the major mTOR protein complexes including total and phosphorylated mTOR (that nucleates the two distinct protein complexes mTORC1 and mTORC2), raptor (specific core regulatory protein of the mTORC1 complex) or rictor (specific core regulatory protein of the mTORC2 complex). Obtained data show a significant decrease in the expression of rictor in the hypothalamus of 6-month-old Thy1-aSYN mice (67.97 ± 9.68 vs. 100 ± 3.66%; *p* < 0.01), suggesting that the mTORC2 pathway is deregulated in 6-month-old Thy1-aSYN mice (**Figure 6B**). Given that rictor plays an important role in autophagy induction and that alpha-synuclein is known to alter autophagy signals, the levels of key autophagy-related proteins LC3B and p62 were assessed but showed no significant changes between Thy1-aSYN and WT mice (89.68 ± 4.96 vs. 100 ± 5.76%; *p* = 0.27 and 102.9 ± 11.47 vs. 100 ± 13.71%; *p* = 0.64, respectively).

**FIGURE 7:**
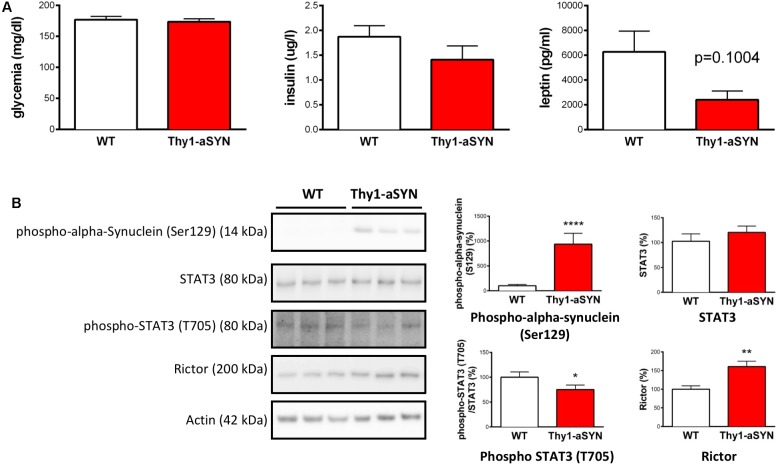
Peripheral hormones and central hypothalamic involvement in 3-month-old animals. **(A)** 3-month-old Thy1-aSYN and WT mice showed no significant difference in blood glucose and plasmatic insulin and leptin levels (WT: *n* = 16, Thy1-aSYN: *n* = 11). **(B)** The left images show representative bands for each target protein, and the results are quantified in the right graphs (WT: *n* = 16, Thy1-aSYN: *n* = 13). Thy1-aSYN mice displayed increased levels of alpha-synuclein phosphorylation at serine 129, as well as decreased STAT3 phosphorylation at tyrosine 705 and increased rictor protein levels. Mann–Whitney *U*-test; ^∗^*p* < 0.05, ^∗∗^*p* < 0.01, and ^∗∗∗∗^*p* < 0.0001 vs. WT mice.

Finally, we aimed to see whether the deregulation of markers linked to metabolism was observed at an earlier time-point in the Thy1-aSYN mouse model. We therefore compared the phenotype of 3-month-old transgenic and WT animals for both peripheral and central markers previously identified as deregulated. Our results show no change in blood glucose levels and insulinemia and a non-statically decrease of the plasmatic levels of leptin in 3-month-old Thy1-aSYN mice compared to controls (2416 ± 699 vs. 6273 ± 1670 pg/ml; *p* = 0.1004) (**Figure 7A**). They moreover evidence in 3-month-old Thy1-aSYN mice increased levels of alpha-synuclein phosphorylated at serine 129 (935.5 ± 218.2 vs. 100 ± 26.60%; *p* < 0.0001) together with a decrease in the phosphorylation of STAT3 at tyrosine 705 (75.24 ± 9.06 vs. 100 ± 10.74%; *p* < 0.05) and an increase in rictor protein levels (160.6 ± 14.48 vs. 100 ± 9.08%; *p* < 0.01) (**Figure 7B**).

## Discussion

The present study aimed to characterize the metabolic phenotype of the Thy1-aSYN mouse model based on the overexpression of full-length, Human, WT alpha-synuclein. The Thy1-aSYN mouse model reproduces several features evoking sporadic PD, such as alpha-synuclein accumulation in brain regions including the substantia nigra. Later in life - at 14 months of age - these mice lose 40% of striatal dopamine and show sensory-motor deficits that, as in Humans, are partially reversed by L-dopa (Lam et al., 2011). This pathological phenotype may be slower than that of mice that overexpress alpha-synuclein carrying mutations found in patients with rare familial forms of PD (i.e., A53T, A30P) (Chesselet and Richter, 2011). In the present study, we characterized the transgenic mice at an age preceding any dopamine loss and severe sensory-motor deficits (Chesselet et al., 2012) in an attempt to improve our understanding at an earlier phase of the disorder. Consistent with previous publications (Fleming et al., 2004; Lam et al., 2011) we report motor deficits in the challenging beam traversal test, therefore supporting the reproducibility of the Thy1-aSYN phenotype. Our data show for the first time that 6-month-old Thy1-aSYN mice display loss of adiposity, altered feeding behavior, decreased EE, as well as deregulation of peripheral hormones and hypothalamic signaling pathways known to be critically implicated in the regulation of energy homeostasis.

### Body Weight and Body Composition of Thy1-aSYN Mice

Thy1-aSYN mice showed a decreased body weight observable from 3 months of age compared to age-matched WT mice, and the difference increased with age. Body composition analysis in 6-month-old animals revealed that Thy1-aSYN mice had lower subcutaneous and visceral fat mass, but no change in lean mass. It has to be noted that Human prospective studies report that PD patients can show decreased body weights several years (2–5 years) before the disease is diagnosed (Durrieu et al., 1992; Chen et al., 2003; Cheshire and Wszolek, 2005; Sharma and Lewis, 2017), after which their average body weights decline. Anthropometric studies show that this weight loss is essentially associated with reduced body fat mass, but not with muscle loss (Markus et al., 1993; Beyer et al., 1995; Lorefalt et al., 2004, 2009). Therefore, the phenotype of the Thy1-aSYN mouse model recapitulates several features reported in PD patients. Because PD patients are at higher risk of reduced bone mineral density and fractures (Torsney et al., 2014), we aimed to measure bone mineral density in the Thy1-aSYN and WT mice, showing no difference between genotypes.

### Altered Feeding Behavior and Energy Metabolism

When individually housed in metabolic cages, 6-month-old transgenic mice showed a decrease in food consumption compared to control mice. This was associated with an altered meal pattern resulting in a smaller meal size. Decreased food consumption and altered meal pattern could arise from a number of factors observed in this transgenic mouse model. First, Thy1-aSYN mice exhibit early and sustained olfactory detection and discrimination deficits without total loss of olfaction, as it is usually reported in patients (Fleming et al., 2008). Olfaction influences food intake (Soria-Gomez et al., 2014) and therefore, such olfactory deficiencies might contribute to a lower food consumption of Thy1-aSYN mice. Second, Thy1-aSYN mice exhibit increased anxiety, that is frequently experienced by PD patients (Mouren et al., 1983). Various studies have associated stress and anxiety to alteration in food intake and body weight (Rivest et al., 1989; Dunn and Berridge, 1990) and this could impact the feeding behavior in our study. Third, Thy1-aSYN mice show reductions in fecal pellet output when exposed to a novelty stress from 2.5–3 months of age, as well as alpha-synuclein accumulation in the colonic myenteric ganglia as reported in 7–8-month-old animals (Wang et al., 2012). Such gastro-intestinal alterations could contribute to weight loss in transgenic animals both by altering their food intake behavior and by causing malabsorption. Thy1-aSYN mice displayed a decreased EE compared to controls, thus arguing against the possibility that an EE dysregulation could contribute to weight loss in our model. Our observations are consistent with the report that weight loss in PD occurs regardless to changes in daily EE (Delikanaki-Skaribas et al., 2009). Analyses of oxygen consumption and RER analyses did not evidence significant changes between transgenic and WT mice. However, they revealed that EE, oxygen consumption and RER were significantly decreased during the light cycle compared to the dark cycle in WT animals but no such differences were observed in Thy1-aSYN mice. This suggests that Thy1-aSYN mice could display alterations in temporal rhythms in energy metabolism and this could be linked to deficits in circadian-regulated behavior (Kudo et al., 2011).

### Hyperactivity, Dopamine, and Face-Validity of the Thy1-aSYN Model

Behavioral characterization of 6-month-old transgenic mice showed an increased activity for both horizontal and vertical movements, in line with previous reports of increased open-field activity of Thy1-aSYN mice at 7 months (Lam et al., 2011). Increased activity of Thy1-aSYN mice has to be considered along with the increased extracellular striatal dopamine reported in the Thy1-aSyn mice at 6 months of age and should be distinguished of the later decreased locomotion observed in 14-month-old Thy1-aSYN mice, when striatal dopamine is significantly lost (Lam et al., 2011; Chesselet et al., 2012). Interestingly, an increased activity has also been reported for other mouse models of PD. For instance, knock-in mice bearing the G2019S LRRK2 mutation (a frequent cause of familial PD) have a hyperactive phenotype in the open field test (Longo et al., 2014). Similarly, MitoPark mice (in which the mitochondrial transcription factor Tfam is selectively removed in midbrain dopamine neurons) display at 6 weeks higher activity scores at both horizontal and vertical movements than control mice, while this hyperactivity is reversed to bradykinesia at 12 weeks of age, when dopamine levels get significantly lower in their striatum (Galter et al., 2010). Although it is difficult to extrapolate these data to patients in which levodopa-responsive motor symptoms support the diagnosis, one could hypothesize that early deregulation of dopamine could contribute to presymptomatic motor changes, as suggested in healthy carriers of the LRRK2 G2019S mutation (Mirelman et al., 2011). Noteworthy, the nigrostriatal dopaminergic pathway has also been implicated in feeding (Ungerstedt, 1971; Szczypka et al., 2001) and an excess of dopamine signaling has been reported to inhibit feeding in mice, as demonstrated with non-specific dopamine receptor agonists, DAT inhibitors, or amphetamines (Leibowitz, 1975). As such, changes in dopamine concentration could contribute to hyperactivity, impairments in challenging motor test and feeding abnormalities in 6-month-old Thy1-aSYN mice.

### Deregulation in Hormones Controlling Energy Homeostasis

Research on the mechanisms governing body weight, feeding behavior and energy metabolism has provided insight into complex interactions between peripheral signals and the central nervous system (Schwartz et al., 2000). Both plasma insulin and leptin concentrations are decreased in 6-month-old Thy1-aSYN mice. The leptin hormone is produced by white adipose tissue (Considine et al., 1996). Its level declines in Humans and mice after weight loss and accordingly a lower plasma leptin concentration was reported in PD patients with weight loss (Lorefalt et al., 2009; Fiszer et al., 2010). Hypoleptinemia has also been reported in transgenic mice overexpressing A53T alpha-synuclein (Rothman et al., 2014), as well as in mice overexpressing amyloid precursor protein (Ishii et al., 2014) and in two Huntington’s disease mouse models (Phan et al., 2009) and might therefore appear as a feature frequently associated with pathological protein accumulation in mice. Insulin is secreted from pancreatic beta cells and its circulating concentrations show a positive correlation with body fat mass (Yu and Kim, 2012). This suggests that the declines in leptin and insulin levels are consequences of the changes in body composition in the Thy1-aSYN mice. In turn, the deregulation of these hormones could alter the phenotype of Thy1-aSYN mice in several ways. For instance, insulin increases adiposity, promotes lipid synthesis, and inhibits lipolysis (Baskin et al., 1999). Also, both leptin and insulin were shown to raise EE when administered to rodents (Menendez and Atrens, 1989; Morton et al., 2011), therefore directly linking decline in these hormones and reduction in EE in the Thy1-aSYN mice. Insulin and leptin have also neuroprotective properties and can influence synaptic plasticity. Among other studies, leptin administration rescued dopaminergic neurons, decreased the apomorphine-induced rotational behavior and restored striatal catecholamine levels in the unilateral 6-hydroxydopamine mouse model of dopaminergic cell death (Weng et al., 2007). Also, insulin induces the expression of the activity-regulated cytoskeleton-associated gene (Chen et al., 2014), an effector immediate early gene critical to protein-synthesis synaptic plasticity (Rosi, 2011). Leptin receptors and insulin receptors are expressed by dopaminergic neurons in midbrain (Figlewicz et al., 2003) and both hormones act directly on these neurons (Hommel et al., 2006; Mebel et al., 2012). Leptin has been shown to modulate dopamine D2 receptor expression in striatum (Pfaffly et al., 2010). Insulin has been shown to depress dopamine concentration in the ventral tegmental area via increasing its reuptake through dopamine transporter (Mebel et al., 2012). Although these results indicate a potential role of leptin and insulin in regulating the action of dopamine in the brain, the exact molecular mechanisms of such actions are yet to be elucidated.

### Alpha-Synuclein and Deregulation of Signaling Pathways Regulating Energy Metabolism in the Hypothalamus of Thy1-aSYN Mice

**FIGURE 8:**
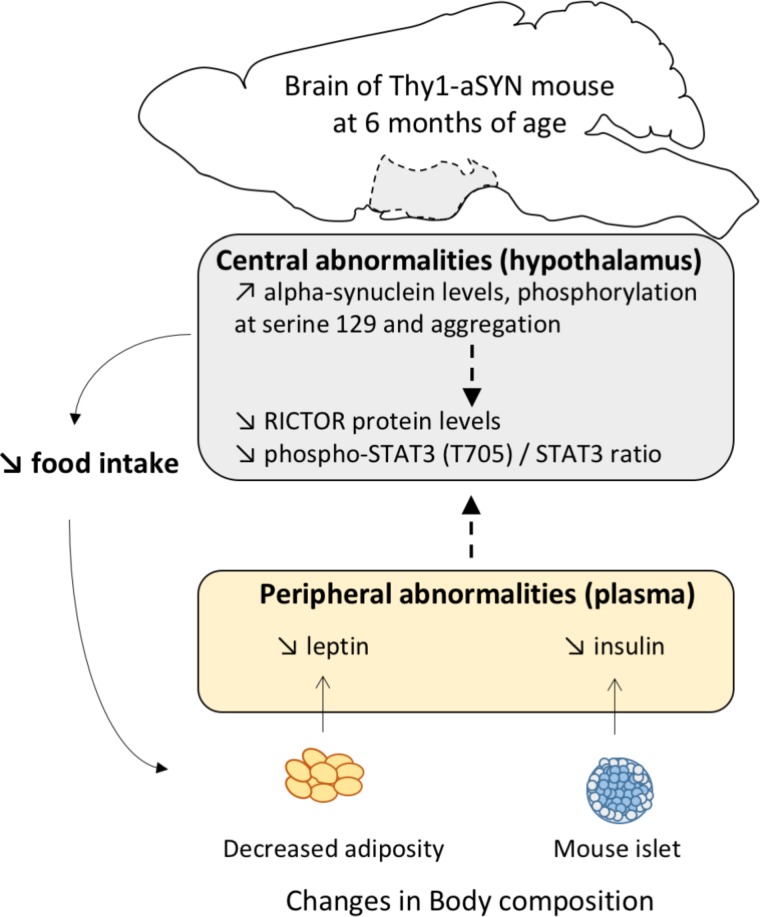
Proposed graphical abstract illustrating the pathways possibly involved in metabolism abnormalities in 6-month-old Thy1-aSYN transgenic mice.

It is now well established that the brain, especially the hypothalamus, maintains body weight homeostasis by effectively adjusting food intake and EE in response to changes in levels of various nutritional status indicators. In PD, alpha-synuclein pathology may spread along neuronal pathways and neuropathological hypothalamic involvement was previously reported (Langston and Forno, 1978; Ansorge et al., 1997), even at preclinical stages (De Pablo-Fernandez et al., 2017). Here, we report for the first time that alpha-synuclein accumulates in the hypothalamus of Thy1-aSYN mice, to levels comparable to those of the *substantia nigra* and the striatum. We also show increased levels of alpha-synuclein phosphorylation at serine 129 and alpha-synuclein aggregation in this brain region. Alpha-synuclein has been suggested to fulfill roles in synaptic function and plasticity and thus it is possible that the alpha-synuclein pathology in the hypothalamus causes functional alterations leading to metabolism abnormalities. Importantly, alpha-synuclein level was also elevated in the plasma of Thy1-aSYN mice compared to WT mice. Although it is not clear how plasma alpha-synuclein levels relate to abnormal aggregated alpha-synuclein in the brain, our results confer to the Thy1-aSYN mouse model a major interest to evaluate the blood as a as a source of neurodegeneration biomarkers. By further analyzing the receptors and protein complexes known to be critically implicated in the regulation of metabolism, we evidenced a decreased phosphorylation at tyrosine 705 of the transcription factor STAT3 in the hypothalamus already present in 3-month-old Thy1-aSYN mice. STAT3 phosphorylation at tyrosine 705 promotes its homodimerization or heterodimerization with other STATs which leads to nucleus translocation and DNA binding. *In vivo* studies have provided evidence indicating that STAT3 activation in the hypothalamus is critical to the regulation of food intake and energy balance, particularly in the functioning of leptin (Bates et al., 2003; Buettner et al., 2006). Thus the downregulation of STAT3 phosphorylation could sign defects in the hypothalamus function in Thy1-aSYN mice. Over the last decade, mTOR complexes 1 and 2 (mTORC1 and mTORC2) have also emerged as critical cellular energy sensors because of their ability to couple hormones (including leptin and insulin) and nutrient signals with the regulation of energy balance and metabolism and activity among others (Haissaguerre et al., 2014). We compared the expression of major mTOR proteins and showed alterations of the protein levels of rictor, a required subunit for mTORC2, with increased protein levels in 3-month-old Thy1-aSYN mice, and decrease protein levels at 6 months of age. We did not observe changes in rictor levels in the *substantia nigra* of 6-month-old Thy1-aSYN (data not shown), suggesting that alpha-synuclein overexpression causes brain region-specific deregulations in our model. Although the function of mTORC2 is not as well-known as the function of mTORC1, it regulates actin polarization and endocytosis (Rispal et al., 2015) and sphingolipid biosynthesis (Roelants et al., 2011). It appears important to further evaluate the role of mTORC2 in these pathways that we and others reported as notably deregulated in PD (Mutez et al., 2014; Dijkstra et al., 2015). Thus, considering the pivotal roles of the hypothalamus in regulating feeding behavior and energy metabolism, it is reasonable to speculate that the STAT3/mTORC2 alterations could contribute to the metabolic phenotype of Thy1-aSYN mice (**Figure 8**).

## Conclusion

In conclusion, our data demonstrate a link between alpha-synuclein overexpression and metabolic abnormalities including decreased body weight and adiposity, altered feeding behavior and decreased EE together with hypoleptinemia and hypoinsulinemia in the Thy1-aSYN mouse model. They moreover raise the possibility that alterations of the STAT3/mTORC2 signaling pathways in the hypothalamus of Thy1-aSYN transgenic mice could contribute to the disruption of their feeding behavior and energy metabolism. This emphasizes the need to better characterize the hypothalamus molecular dysregulation in PD to understand the etiology of unintended body weight change in this disease. Future studies are particularly needed to evaluate the interest of the proteins linked to STAT3 and mTORC2 signaling as biomarkers for PD and to test whether strategies aimed to normalize energy metabolism would interfere with alpha-synuclein-linked pathology.

## Author Contributions

ECu, MM, CL, WS and ASt contributed to the research project execution and to the data and statistical analyses. CV-M, OV, and ECu contributed to the project conception and to the manuscript reviewing. ASt, ASa, KS, TC, PS, M-AB, HC helped with the project execution. ECa supervised the use of the metabolic cages. AL helped with the meal pattern analysis. EM, AD, and BG revised the manuscript providing expertise in PD and cell signaling. M-CC-H contributed to the project conception and organization and edited the manuscript providing expertise in molecular neurodegeneration in PD. KB contributed to the project conception, organization and execution, to the statistical analyses and predominantly contributed to the writing of the article. All authors read and approved the final manuscript.

## Conflict of Interest Statement

The authors declare that the research was conducted in the absence of any commercial or financial relationships that could be construed as a potential conflict of interest.

## References

[B1] Aiello M. Eleopra R. Foroni F. Rinaldo S. Rumiati R. I. (2017). Weight gain after STN-DBS: the role of reward sensitivity and impulsivity. *Cortex* 92 150–161. 10.1016/j.cortex.2017.04.005 28494345

[B2] Ansorge O. Daniel S. E. Pearce R. K. (1997). Neuronal loss and plasticity in the supraoptic nucleus in Parkinson’s disease. *Neurology* 49 610–613. 10.1212/WNL.49.2.610 9270609

[B3] Bannier S. Montaurier C. Derost P. P. Ulla M. Lemaire J. J. Boirie Y. (2009). Overweight after deep brain stimulation of the subthalamic nucleus in Parkinson disease: long term follow-up. *J. Neurol. Neurosurg. Psychiatry* 80 484–488. 10.1136/jnnp.2008.158576 19060023

[B4] Baskin D. G. Figlewicz Lattemann D Seeley R. J. Woods S. C. Porte D Jr Schwartz M. W. (1999). Insulin and leptin: dual adiposity signals to the brain for the regulation of food intake and body weight. *Brain Res.* 848 114–123. 10.1016/S0006-8993(99)01974-5 10612703

[B5] Bates S. H. Stearns W. H. Dundon T. A. Schubert M. Tso A. W. Wang Y. (2003). STAT3 signalling is required for leptin regulation of energy balance but not reproduction. *Nature* 421 856–859. 10.1038/nature01388 12594516

[B6] Beyer P. L. Palarino M. Y. Michalek D. Busenbark K. Koller W. C. (1995). Weight change and body composition in patients with Parkinson’s disease. *J. Am. Diet. Assoc.* 95 979–983. 10.1016/S0002-8223(95)00269-3 7657912

[B7] Bezard E. Dovero S. Prunier C. Ravenscroft P. Chalon S. Guilloteau D. (2001). Relationship between the appearance of symptoms and the level of nigrostriatal degeneration in a progressive 1-methyl-4-phenyl-1,2,3,6-tetrahydropyridine-lesioned macaque model of Parkinson’s disease. *J. Neurosci.* 21 6853–6861. 10.1523/JNEUROSCI.21-17-06853.2001 11517273 PMC6763089

[B8] Bridi J. C. Hirth F. (2018). Mechanisms of alpha-synuclein induced synaptopathy in Parkinson’s Disease. *Front. Neurosci.* 12:80. 10.3389/fnins.2018.00080 29515354 PMC5825910

[B9] Buettner C. Pocai A. Muse E. D. Etgen A. M. Myers MG Jr Rossetti L. (2006). Critical role of STAT3 in leptin’s metabolic actions. *Cell Metab.* 4 49–60. 10.1016/j.cmet.2006.04.014 16814732 PMC3638026

[B10] Butler A. A. Kozak L. P. (2010). A recurring problem with the analysis of energy expenditure in genetic models expressing lean and obese phenotypes. *Diabetes Metab. Res. Rev.* 59 323–329. 10.2337/db09-1471 20103710 PMC2809965

[B11] Chartier-Harlin M. C. Kachergus J. Roumier C. Mouroux V. Douay X. Lincoln S. (2004). Alpha-synuclein locus duplication as a cause of familial Parkinson’s disease. *Lancet* 364 1167–1169. 10.1016/S0140-6736(04)17103-1 15451224

[B12] Chen H. Zhang S. M. Hernan M. A. Willett W. C. Ascherio A. (2003). Weight loss in Parkinson’s disease. *Ann. Neurol.* 53 676–679. 10.1002/ana.10577 12731005

[B13] Chen T. J. Wang D. C. Hung H. S. Ho H. F. (2014). Insulin can induce the expression of a memory-related synaptic protein through facilitating AMPA receptor endocytosis in rat cortical neurons. *Cell Mol. Life Sci.* 71 4069–4080. 10.1007/s00018-014-1620-5 24705985 PMC11113657

[B14] Cheshire W. P. Jr. Wszolek Z. K. (2005). Body mass index is reduced early in Parkinson’s disease. *Parkinsonism Relat. Disord.* 11 35–38. 10.1016/j.parkreldis.2004.07.001 15619460

[B15] Chesselet M. F. Richter F. (2011). Modelling of Parkinson’s disease in mice. *Lancet Neurol.* 10 1108–1118. 10.1016/S1474-4422(11)70227-7 22094131

[B16] Chesselet M. F. Richter F. Zhu C. Magen I. Watson M. B. Subramaniam S. R. (2012). A progressive mouse model of Parkinson’s disease: the Thy1-aSyn (“Line 61”) mice. *Neurotherapeutics* 9 297–314. 10.1007/s13311-012-0104-2 22350713 PMC3337020

[B17] Considine R. V. Sinha M. K. Heiman M. L. Kriauciunas A. Stephens T. W. Nyce M. R. (1996). Serum immunoreactive-leptin concentrations in normal-weight and obese humans. *N. Engl. J. Med.* 334 292–295. 10.1056/NEJM199602013340503 8532024

[B18] Cumming K. Macleod A. D. Myint P. K. Counsell C. E. (2017). Early weight loss in parkinsonism predicts poor outcomes: evidence from an incident cohort study. *Neurology* 89 2254–2261. 10.1212/WNL.0000000000004691 29079685 PMC5705250

[B19] De Pablo-Fernandez E. Courtney R. Holton J. L. Warner T. T. (2017). Hypothalamic alpha-synuclein and its relation to weight loss and autonomic symptoms in Parkinson’s disease. *Mov. Disord.* 32 296–298. 10.1002/mds.26868 27892607

[B20] Delikanaki-Skaribas E. Trail M. Wong W. W. Lai E. C. (2009). Daily energy expenditure, physical activity, and weight loss in Parkinson’s disease patients. *Mov. Disord.* 24 667–671. 10.1002/mds.22372 19117356

[B21] Dijkstra A. A. Ingrassia A. de Menezes R. X. van Kesteren R. E. Rozemuller A. J. Heutink P. (2015). Evidence for immune response, axonal dysfunction and reduced endocytosis in the substantia nigra in early stage parkinson’s disease. *PLoS One* 10:e0128651. 10.1371/journal.pone.0128651 26087293 PMC4472235

[B22] Dunn A. J. Berridge C. W. (1990). Physiological and behavioral responses to corticotropin-releasing factor administration: is CRF a mediator of anxiety or stress responses? *Brain Res. Brain Res. Rev.* 15 71–100. 10.1016/0165-0173(90)90012-D 1980834

[B23] Durrieu G. Me L. L. Rascol O. Senard J. M. Rascol A. Montastruc J. L. (1992). Parkinson’s disease and weight loss: a study with anthropometric and nutritional assessment. *Clin. Auton. Res.* 2 153–157. 10.1007/BF01818955 1498561

[B24] Fereshtehnejad S. M. Ghazi L. Sadeghi M. Khaefpanah D. Shahidi G. A. Delbari A. (2014). Prevalence of malnutrition in patients with Parkinson’s disease: a comparative study with healthy controls using Mini Nutritional Assessment (MNA) questionnaire. *J. Parkinsons Dis.* 4 473–481. 10.3233/JPD-130323 24718272

[B25] Figlewicz D. P. Evans S. B. Murphy J. Hoen M. Baskin D. G. (2003). Expression of receptors for insulin and leptin in the ventral tegmental area/substantia nigra (VTA/SN) of the rat. *Brain Res.* 964 107–115. 10.1016/S0006-8993(02)04087-8 12573518

[B26] Fiszer U. Michalowska M. Baranowska B. Wolinska-Witort E. Jeske W. Jethon M. (2010). Leptin and ghrelin concentrations and weight loss in Parkinson’s disease. *Acta Neurol. Scand.* 121 230–236. 10.1111/j.1600-0404.2009.01185.x 20028343

[B27] Fleming S. M. Ekhator O. R. Ghisays V. (2013). Assessment of sensorimotor function in mouse models of Parkinson’s disease. *J. Vis. Exp.* 76:e50303. 10.3791/50303 23851663 PMC3727502

[B28] Fleming S. M. Salcedo J. Fernagut P. O. Rockenstein E. Masliah E. Levine M. S. (2004). Early and progressive sensorimotor anomalies in mice overexpressing wild-type human alpha-synuclein. *J. Neurosci.* 24 9434–9440. 10.1523/JNEUROSCI.3080-04.2004 15496679 PMC6730110

[B29] Fleming S. M. Tetreault N. A. Mulligan C. K. Hutson C. B. Masliah E. Chesselet M. F. (2008). Olfactory deficits in mice overexpressing human wildtype alpha-synuclein. *Eur. J. Neurosci.* 28 247–256. 10.1111/j.1460-9568.2008.06346.x 18702696 PMC3108548

[B30] Galter D. Pernold K. Yoshitake T. Lindqvist E. Hoffer B. Kehr J. (2010). MitoPark mice mirror the slow progression of key symptoms and L-DOPA response in Parkinson’s disease. *Genes Brain Behav.* 9 173–181. 10.1111/j.1601-183X.2009.00542.x 20002202 PMC4154513

[B31] Haissaguerre M. Saucisse N. Cota D. (2014). Influence of mTOR in energy and metabolic homeostasis. *Mol. Cell. Endocrinol.* 397 67–77. 10.1016/j.mce.2014.07.015 25109278

[B32] Hassouna R. Labarthe A. Zizzari P. Videau C. Culler M. Epelbaum J. (2013). Actions of agonists and antagonists of the ghrelin/GHS-R pathway on GH secretion, appetite, and cFos activity. *Front. Endocrinol. (Lausanne)* 4:25. 10.3389/fendo.2013.00025 23515849 PMC3600614

[B33] Hommel J. D. Trinko R. Sears R. M. Georgescu D. Liu Z. W. Gao X. B. (2006). Leptin receptor signaling in midbrain dopamine neurons regulates feeding. *Neuron* 51 801–810. 10.1016/j.neuron.2006.08.023 16982424

[B34] Ibanez P. Bonnet A. M. Debarges B. Lohmann E. Tison F. Pollak P. (2004). Causal relation between alpha-synuclein gene duplication and familial Parkinson’s disease. *Lancet* 364 1169–1171. 10.1016/S0140-6736(04)17104-3 15451225

[B35] Ishii M. Wang G. Racchumi G. Dyke J. P. Iadecola C. (2014). Transgenic mice overexpressing amyloid precursor protein exhibit early metabolic deficits and a pathologically low leptin state associated with hypothalamic dysfunction in arcuate neuropeptide Y neurons. *J. Neurosci.* 34 9096–9106. 10.1523/JNEUROSCI.0872-14.2014 24990930 PMC4078086

[B36] Jorgensen H. U. Werdelin L. Lokkegaard A. Westerterp K. R. Simonsen L. (2012). Free-living energy expenditure reduced after deep brain stimulation surgery for Parkinson’s disease. *Clin. Physiol. Funct. Imaging* 32 214–220. 10.1111/j.1475-097X.2011.01079.x 22487156

[B37] Kaiyala K. J. Morton G. J. Leroux B. G. Ogimoto K. Wisse B. Schwartz M. W. (2010). Identification of body fat mass as a major determinant of metabolic rate in mice. *Diabetes Metab. Res. Rev.* 59 1657–1666. 10.2337/db09-1582 20413511 PMC2889765

[B38] Kudo T. Loh D. H. Truong D. Wu Y. Colwell C. S. (2011). Circadian dysfunction in a mouse model of Parkinson’s disease. *Exp. Neurol.* 232 66–75. 10.1016/j.expneurol.2011.08.003 21864527

[B39] Lam H. A. Wu N. Cely I. Kelly R. L. Hean S. Richter F. (2011). Elevated tonic extracellular dopamine concentration and altered dopamine modulation of synaptic activity precede dopamine loss in the striatum of mice overexpressing human alpha-synuclein. *J. Neurosci. Res.* 89 1091–1102. 10.1002/jnr.22611 21488084 PMC4755488

[B40] Langston J. W. Forno L. S. (1978). The hypothalamus in Parkinson disease. *Ann. Neurol.* 3 129–133. 10.1002/ana.410030207 350130

[B41] Leibowitz S. F. (1975). Amphetamine: possible site and mode of action for producing anorexia in the rat. *Brain Res.* 84 160–167. 10.1016/0006-8993(75)90811-2 1053933

[B42] Longo F. Russo I. Shimshek D. R. Greggio E. Morari M. (2014). Genetic and pharmacological evidence that G2019S LRRK2 confers a hyperkinetic phenotype, resistant to motor decline associated with aging. *Neurobiol. Dis.* 71 62–73. 10.1016/j.nbd.2014.07.013 25107341 PMC4194318

[B43] Lorefalt B. Ganowiak W. Palhagen S. Toss G. Unosson M. Granerus A. K. (2004). Factors of importance for weight loss in elderly patients with Parkinson’s disease. *Acta Neurol. Scand.* 110 180–187. 10.1111/j.1600-0404.2004.00307.x 15285776

[B44] Lorefalt B. Toss G. Granerus A. K. (2009). Weight loss, body fat mass, and leptin in Parkinson’s disease. *Mov. Disord.* 24 885–890. 10.1002/mds.22466 19199361

[B45] Ma K. Xiong N. Shen Y. Han C. Liu L. Zhang G. (2018). Weight loss and malnutrition in patients with parkinson’s disease: current knowledge and future prospects. *Front. Aging Neurosci.* 10:1 10.3389/fnagi.2018.00001 PMC5780404 29403371

[B46] Maraganore D. M. de Andrade M. Elbaz A. Farrer M. J. Ioannidis J. P. Kruger R. (2006). Collaborative analysis of alpha-synuclein gene promoter variability and Parkinson disease. *JAMA* 296 661–670. 10.1001/jama.296.6.661 16896109

[B47] Markus H. S. Cox M. Tomkins A. M. (1992). Raised resting energy expenditure in Parkinson’s disease and its relationship to muscle rigidity. *Clin. Sci. (Lond.)* 83 199–204. 10.1042/cs0830199 1327636

[B48] Markus H. S. Tomkins A. M. Stern G. M. (1993). Increased prevalence of undernutrition in Parkinson’s disease and its relationship to clinical disease parameters. *J. Neural Transm. Park Dis. Dement Sect.* 5 117–125. 10.1007/BF02251202 8333907

[B49] Mebel D. M. Wong J. C. Dong Y. J. Borgland S. L. (2012). Insulin in the ventral tegmental area reduces hedonic feeding and suppresses dopamine concentration via increased reuptake. *Eur. J. Neurosci.* 36 2336–2346. 10.1111/j.1460-9568.2012.08168.x 22712725 PMC5239666

[B50] Menendez J. A. Atrens D. M. (1989). Insulin increases energy expenditure and respiratory quotient in the rat. *Pharmacol. Biochem. Behav.* 34 765–768. 10.1016/0091-3057(89)90272-4 2516326

[B51] Mequinion M. Caron E. Zgheib S. Stievenard A. Zizzari P. Tolle V. (2015). Physical activity: benefit or weakness in metabolic adaptations in a mouse model of chronic food restriction? *Am. J. Physiol. Endocrinol. Metab.* 308 E241–E255. 10.1152/ajpendo.00340.2014 25465889

[B52] Mirelman A. Gurevich T. Giladi N. Bar-Shira A. Orr-Urtreger A. Hausdorff J. M. (2011). Gait alterations in healthy carriers of the LRRK2 G2019S mutation. *Ann. Neurol.* 69 193–197. 10.1002/ana.22165 21280089

[B53] Morton G. J. Kaiyala K. J. Fisher J. D. Ogimoto K. Schwartz M. W. Wisse B. E. (2011). Identification of a physiological role for leptin in the regulation of ambulatory activity and wheel running in mice. *Am. J. Physiol. Endocrinol. Metab.* 300 E392–E401. 10.1152/ajpendo.00546.2010 21062956 PMC3043625

[B54] Mouren P. Poinso Y. Oppenheim G. Mouren A. Nguyen Quang M. (1983). [Personality of the parkinsonian, clinical and psychometric approach]. *Ann. Med. Psychol. (Paris)* 141 153–167. 6614722

[B55] Mutez E. Nkiliza A. Belarbi K. de Broucker A. Vanbesien-Mailliot C. Bleuse S. (2014). Involvement of the immune system, endocytosis and EIF2 signaling in both genetically determined and sporadic forms of Parkinson’s disease. *Neurobiol. Dis.* 63 165–170. 10.1016/j.nbd.2013.11.007 24269915

[B56] Pak K. Shin H. K. Kim E. J. Lee J. H. Lyoo C. H. Son J. (2018). Weight loss is associated with rapid striatal dopaminergic degeneration in Parkinson’s disease. *Parkinsonism Relat. Disord.* 51 67–72. 10.1016/j.parkreldis.2018.02.044 29510907

[B57] Pfaffly J. Michaelides M. Wang G. J. Pessin J. E. Volkow N. D. Thanos P. K. (2010). Leptin increases striatal dopamine D2 receptor binding in leptin-deficient obese (ob/ob) mice. *Synapse* 64 503–510. 10.1002/syn.20755 20175225 PMC2873172

[B58] Phan J. Hickey M. A. Zhang P. Chesselet M. F. Reue K. (2009). Adipose tissue dysfunction tracks disease progression in two Huntington’s disease mouse models. *Hum. Mol. Genet.* 18 1006–1016. 10.1093/hmg/ddn428 19124532 PMC2649017

[B59] Polymeropoulos M. H. Lavedan C. Leroy E. Ide S. E. Dehejia A. Dutra A. (1997). Mutation in the alpha-synuclein gene identified in families with Parkinson’s disease. *Science* 276 2045–2047. 10.1126/science.276.5321.2045 9197268

[B60] Rispal D. Eltschinger S. Stahl M. Vaga S. Bodenmiller B. Abraham Y. (2015). Target of rapamycin complex 2 regulates actin polarization and endocytosis via multiple pathways. *J. Biol. Chem.* 290 14963–14978. 10.1074/jbc.M114.627794 25882841 PMC4463442

[B61] Rivest S. Deshaies Y. Richard D. (1989). Effects of corticotropin-releasing factor on energy balance in rats are sex dependent. *Am. J. Physiol.* 257(6 Pt 2), R1417–R1422. 10.1152/ajpregu.1989.257.6.R1417 2604001

[B62] Roelants F. M. Breslow D. K. Muir A. Weissman J. S. Thorner J. (2011). Protein kinase Ypk1 phosphorylates regulatory proteins Orm1 and Orm2 to control sphingolipid homeostasis in *Saccharomyces cerevisiae*. *Proc. Natl. Acad. Sci. U.S.A.* 108 19222–19227. 10.1073/pnas.1116948108 22080611 PMC3228448

[B63] Rosi S. (2011). Neuroinflammation and the plasticity-related immediate-early gene Arc. *Brain Behav. Immun.* 25(Suppl. 1), S39–S49. 10.1016/j.bbi.2011.02.003 21320587 PMC3098296

[B64] Rothman S. M. Griffioen K. J. Fishbein K. W. Spencer R. G. Makrogiannis S. Cong W. N. (2014). Metabolic abnormalities and hypoleptinemia in alpha-synuclein A53T mutant mice. *Neurobiol. Aging* 35 1153–1161. 10.1016/j.neurobiolaging.2013.10.088 24239384 PMC3946232

[B65] Schapira A. H. V. Chaudhuri K. R. Jenner P. (2017). Non-motor features of Parkinson disease. *Nat. Rev. Neurosci.* 18 435–450. 10.1038/nrn.2017.62 28592904

[B66] Schwartz M. W. Woods S. C. Porte D. Jr. Seeley R. J. Baskin D. G. (2000). Central nervous system control of food intake. *Nature* 404 661–671. 10.1038/35007534 10766253

[B67] Sharma J. C. Lewis A. (2017). Weight in Parkinson’s disease: phenotypical significance. *Int. Rev. Neurobiol.* 134 891–919. 10.1016/bs.irn.2017.04.011 28805588

[B68] Sharma J. C. Vassallo M. (2014). Prognostic significance of weight changes in Parkinson’s disease: the Park-weight phenotype. *Neurodegener. Dis. Manag.* 4 309–316. 10.2217/nmt.14.25 25313987

[B69] Simon-Sanchez J. Schulte C. Bras J. M. Sharma M. Gibbs J. R. Berg D. (2009). Genome-wide association study reveals genetic risk underlying Parkinson’s disease. *Nat. Genet.* 41 1308–1312. 10.1038/ng.487 19915575 PMC2787725

[B70] Singleton A. B. Farrer M. Johnson J. Singleton A. Hague S. Kachergus J. (2003). Alpha-synuclein locus triplication causes Parkinson’s disease. *Science* 302:841 10.1126/science.1090278302/5646/841 14593171

[B71] Soria-Gomez E. Bellocchio L. Reguero L. Lepousez G. Martin C. Bendahmane M. (2014). The endocannabinoid system controls food intake via olfactory processes. *Nat. Neurosci.* 17 407–415. 10.1038/nn.3647 24509429

[B72] Szczypka M. S. Kwok K. Brot M. D. Marck B. T. Matsumoto A. M. Donahue B. A. (2001). Dopamine production in the caudate putamen restores feeding in dopamine-deficient mice. *Neuron* 30 819–828. 10.1016/S0896-6273(01)00319-1 11430814

[B73] Torsney K. M. Noyce A. J. Doherty K. M. Bestwick J. P. Dobson R. Lees A. J. (2014). Bone health in Parkinson’s disease: a systematic review and meta-analysis. *J. Neurol. Neurosurg. Psychiatry* 85 1159–1166. 10.1136/jnnp-2013-307307 24620034 PMC4173751

[B74] Uc E. Y. Struck L. K. Rodnitzky R. L. Zimmerman B. Dobson J. Evans W. J. (2006). Predictors of weight loss in Parkinson’s disease. *Mov. Disord.* 21 930–936. 10.1002/mds.20837 16534756

[B75] Ungerstedt U. (1971). Adipsia and aphagia after 6-hydroxydopamine induced degeneration of the nigro-striatal dopamine system. *Acta Physiol. Scand. Suppl.* 367 95–122. 10.1111/j.1365-201X.1971.tb11001.x 4332694

[B76] van der Marck M. A. Dicke H. C. Uc E. Y. Kentin Z. H. Borm G. F. Bloem B. R. (2012). Body mass index in Parkinson’s disease: a meta-analysis. *Parkinsonism Relat. Disord.* 18 263–267. 10.1016/j.parkreldis.2011.10.016 22100523

[B77] Wang L. Magen I. Yuan P. Q. Subramaniam S. R. Richter F. Chesselet M. F. (2012). Mice overexpressing wild-type human alpha-synuclein display alterations in colonic myenteric ganglia and defecation. *Neurogastroenterol. Motil.* 24 e425–e436. 10.1111/j.1365-2982.2012.01974.x 22779732 PMC3712640

[B78] Weng Z. Signore A. P. Gao Y. Wang S. Zhang F. Hastings T. (2007). Leptin protects against 6-hydroxydopamine-induced dopaminergic cell death via mitogen-activated protein kinase signaling. *J. Biol. Chem.* 282 34479–34491. 10.1074/jbc.M705426200 17895242

[B79] Yu J. H. Kim M. S. (2012). Molecular mechanisms of appetite regulation. *Diabetes Metab. J.* 36 391–398. 10.4093/dmj.2012.36.6.391 23275931 PMC3530708

